# Changes in the pathophysiology of viral variants of COVID-19 with a significant reduction in thrombotic events

**DOI:** 10.1590/1806-9282.20241110

**Published:** 2024-12-02

**Authors:** Jose Maria Pereira de Godoy, Gleison Juliano da Silva Russeff

**Affiliations:** 1 Faculty Medicine in São José do Rio Preto, Department Cardiology and Cardiovascular Surgery, Head Vascular Surgery Discipline in Faculty Medicine in São José do Rio Preto and National Council for Research and Development – São José do Rio Preto (SP), Brazil.; 2 Base Hospital, Faculty of Medicine Foundation of Sao Jose do Rio Preto, Ecography Service – São José do Rio Preto (SP), Brazil.

Dear Editor,

COVID-19 was a global challenge in the face of sudden changes that brought another and even greater challenge to the global health system. At a teaching hospital, Hospital de Base de São Jose do Rio Preto, what we believe was the biggest challenge was related to the need to adapt in order to care for more than 9,000 hospitalized patients, with more than 50% in intensive care units. Addressing thrombotic events was one of the problems to be faced, with a large number of patients hospitalized for COVID-19 suffering from deep vein thrombotic events^1–3^.

A total of 9,049 patients with a hypothesis of deep vein thrombosis (DVT) were evaluated during the pandemic from July 2020 to December 2023. There was a significant variation in the positivity of test results between the viral variants compared to the pre-COVID-19 period. In 1999, that is, in the pre-COVID-19 period, test positivity was 32.4%, and during the pandemic, between March 2020 and December 2023, this rose to 30.1%. Throughout this period, there was a higher number of exam requests with many not having well-defined criteria for DVT, suggesting the need for more care when faced with diagnostic doubts.

In the first phase of the pandemic without viral predominance, there were 979 patients with suspected DVT, averaging 139 tests/month, in which 297 (30.3%) of which were positive, with 23.5% of these being associated with COVID-19. When compared with the monthly number of patients requested in 1999, before the pandemic, there were an average of 109 exams/month, with a positivity rate of 32.4% for DVT, giving a statistically significant difference (unpaired t-test p=0.005).

**Figure 1:**
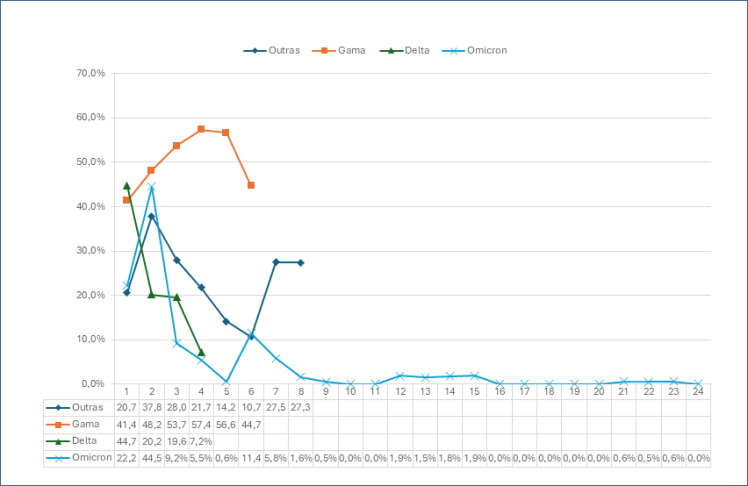
Monthly assessment of deep vein thrombosis.

In the period of the Gamma variant, 1,574 patients were suspected of having DVT with 591 (37.54%) proving to be positive and 60.36% of these instances resulting from COVID-19. In this period, 262 tests were requested per month, a significant increase with respect to requests both prior to COVID-19 and even at the beginning of the pandemic (p<0.05). Regarding the Delta variant, 593 hypotheses of DVT were recorded with 179 (30.18%) being positive and 15.6% associated with COVID-19; around 198 tests were carried out per month. During the Omicron outbreak in 2022 and 2023, there were 4,662 suspected DVT cases of which 1,174 were positive (25.18%), but only 8.6% were associated with COVID-19 in 2022 and 0.6% in 2023. The average number of exam requests per month in 2022 was 185, and in 2023, it was 170 exams (Table 1 and Figure 1).

**Table 1:** Monthly mean number of requests for Doppler exams to evaluate suspected deep vein thrombosis cases and deep vein thrombosis positivity, before COVID-19, at the onset of the pandemic and related to specific variants.

	Exams/month	% Positive exams	% Related to COVID-19
1999	109	32.4%	
COVID—onset	139	30.3%	23.5%
Gamma	262	37.5%	60.36%
Delta	198	30.18%	15.6%
Omicron (2022)	185	27.69%	8.6%
Omicron (2023)	170	27.38%	0.6%

This study shows that there was a greater request for tests during the pandemic, however positivity before COVID-19 was 32.4% and for the Gamma and Delta variants it was 37.5 and 30.18%, respectively, thereafter reducing during the Omicron variant period to around 27.5%. This shows that a misinterpretation may occur when the total volume of tests is analyzed, before COVID-19 with a positivity of (32.4%) and during the pandemic (30.01%). Analyzing the viral variants, we detected that with the Gamma and Delta variants, the overall number of positive cases was no different compared to the pre-COVID-19 period; however, in the Omicron period, this reduced significantly to 27.5%.

The justification for the reduction in positivity lies in the greater number of D-dimer tests requested in Brazil due to patients’ anxiety regarding history of thrombosis linked to COVID-19, which often led doctors to request more tests based solely on elevated D-dimer levels in asymptomatic patients.

The greatest contribution of this study is to show the change in the pathophysiological involvement of COVID-19, where in the initial phase thrombotic events were one of the biggest challenges in treating patients. The Omicron period brought an important pathophysiological change with the type of complication shifting to infectious complaints.
